# Supplementary material for: Shape Dependence of Silver-Nanoparticle-Mediated Synthesis of Gold Nanoclusters with Small Molecules as Capping Ligands
Source: Nanomaterials (Basel). 2023 Aug 14;13(16):2338. doi: 10.3390/nano13162338 (PMC10457754; doi:10.3390/nano13162338)
Supplement: Supplementary file 1 [file nanomaterials-13-02338-s001.zip › nanomaterials-2520203-supplementary.pdf]

Shape Dependence of Silver-Nanoparticle-Mediated Synthesis of Gold Nanoclusters with Small Molecules as Capping Ligands

Cheng-Yeh Chang †, Yi-Ru Wu †, Tzu-Hsien Tseng †, Jun-Hao Su, Yu-Shan Wang, Fang-Yi Jen, Bo-Ru Chen, Cheng-Liang Huang and Jui-Chang Chen *

Department of Applied Chemistry, National Chiayi University, Chiayi City 600355, Taiwan; sss1072727@gmail.com (C.-Y.C.); jerry19970228@gmail.com (Y.-R.W.); tzuhome50@gmail.com (T.-H.T.); andsonhao@gmail.com (J.-H.S.); wang30534@gmail.com (Y.-S.W.); sabrinajengg@gmail.com (F.-Y.J.); s1090251@mail.ncyu.edu.tw (B.-R.C.); clhuang@mail.ncyu.edu.tw (C.-L.H.)

* Correspondence: chenjc@mail.ncyu.edu.tw; Tel.: +886-5-2717968; Fax: +886-5-2717901

† These authors contributed equally to this work.

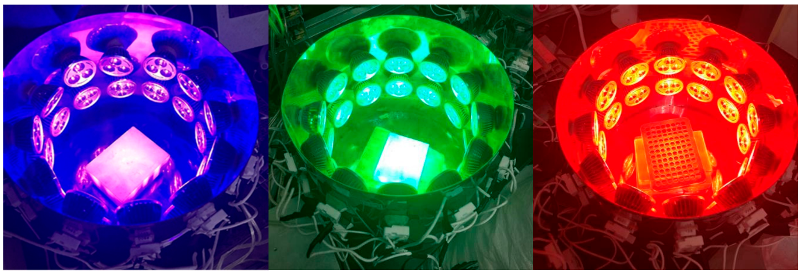

Figure S1. The setups for LED irradiation to generate AgNPs. The cabinet, containing 24 LEDs with 405 nm wavelengths (violet LED, left panel), 525 nm wavelengths (green LED, center panel), and 630 nm wavelengths (red LED, right panel), were used for uniform light intensity (0.01 W/cm^2), surrounding a 50-mL reaction vial.

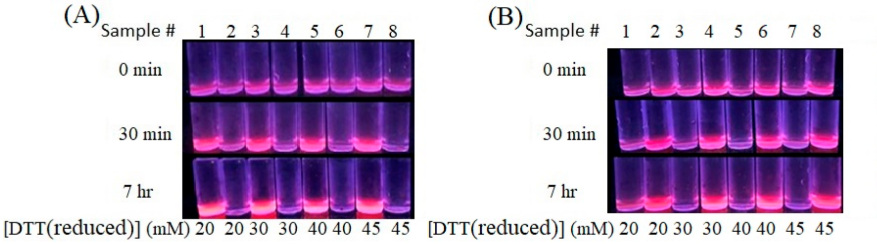

Figure S2. Synthesis of DTT-Au NCs using 0.4 mM $HAuCl_4$ and 4.8 × 10^{-2} mM different shapes of AgNPs. Concentrations of DTT are indicated on the bottom of each figure. The images of aqueous solutions of the as-prepared DTT-Au NCs are taken under UV-box detection. (A) Odd numbers (1, 3, 5, and 7) indicate that QS-AgNPs were used as reductants and even numbers (2, 4, 6, and 8) indicate that T-AgNPs were used as the reductants. (B) Odd numbers indicate S-AgNPs were used as reductants and even numbers indicate that AgNPts were used as the reductants.
